# Comparison of English and American Railway Cars

**Published:** 1882-12

**Authors:** 


### COMPARISION OF ENGLISH AND AMERICAN RAILWAY CARS.

Mr. R. A. Proctor, who has traveled all over this country, and ought to know, if anybody does, what the merits of railway cars are, gives the following in *Knowledge:*

Every one who has traveled much both in Europe and America will agree with Mr. Sala's remark that "our present locked-in, boxed-up, stuffy and narrow compartments are absurd, dangerous and scandalous to us as a nation." Because when railway traveling was first introduced stage coaches were in fashion, the idea which a "slow" railway projector naturally formed was to make a train consist of a number of rather large stage-coaches ; and this arrangement, which was feeble-minded enough then, has remained in vogue for more than half a century.

Let me briefly enumerate a few of the advantages of the American system, and then I will touch on their more or less imaginary disadvantages:

First, you can get on board an American train or leave it when the train is moving pretty fast in perfect safety. I have run after a train and got in the rear car (with a helping hand from a brakeman) when it had attained a rate of certainly twelve miles an hour. I have never left one traveling at that rate, but by the rear car it could be done safely enough—at no worse expense than a sprawl.

Secondly, when on board you can choose any car or any part of any car to sit in ; you can go to the smoking car, if you want to ; or, if you like, you can visit the baggage van to see that your luggage is safe—all when the train is at full speed. I have walked the whole length of a train with both hands occupied by satchels, etc., stopping only when opening and shutting the car doors.

Thirdly, if pressed for time, you can, in nearly all parts of America, go on board without a ticket, and obtain one at the first visit of the conductor.

Fourthly, tickets are attended to while the train is traveling. There is no absurd stoppage at the last station but one and proclamation of "All tickets ready !" but, without delay of any sort, all tickets are collected *en route.*

Fifthly, the travelers by the train form a single community, with a force of conductors, brakemen, porters and luggage men, so that if a disorderly or drunken person gets on board he must behave himself, at the risk of being turned off the train (in bad cases while the train is moving pretty fast, so that his exit is hasty and undignified, yet not unpleasing to those he had thought to annoy).

Sixthly, you generally travel in much more real privacy and comfort than in an English first-class carriage, not secured by a lawless fee to the guard. I used to find quite a rest in a railway journey between my lectures in America, with a little two-seat compartment to myself, all the passengers sitting in similar compartments facing one way ; I could read or reflect undisturbed. Who can say quite as much of an English first-class carriage, if there are two or three passengers on the opposite seats ? It is true that part of this arises from the "stony British stare," which foreigners and Americans find so strange and unpleasant. But "fix it how you will," you can never feel quite so much at ease facing several persons as when all face the same way. On one very special occasion, in America, when I had to travel in an ordinary car for several hours under circumstances which would have made staring excusable enough (not to make a mystery where there need be none, I was one of a wedding party of two), I was struck by the careful courtesy with which a two-seat compartment seemed to be regarded as if it were a private sitting room. I never more thoroughly recognized the intimate courtesy of all Americans toward ladies than I did on that occasion. Of course, when traveling in an American car, a man may be addressed by a fellow passenger more freely than in England. But it is easy to answer pleasantly ; and if the conversation wearies, either to close it or seek another place.

Seventhly, all the carriages are well warmed, and warmed quite safely. I speak without any prejudice in favor of car stoves; for in a railway accident in Missouri I made a much more intimate acquaintance with one than I cared for, and shall carry the marks of the encounter to the grave. But one cannot expect stoves to behave well when the car they are intended to warm is pitched over an embankment thirty feet high. Under all the usual conditions of travel they are pefectly safe traveling companions, and many a time and oft I have missed them when shivering in an English first-class carriage despite wraps and the abomination known as a foot-warmer.

Eighthly, in all cars there is a retiring room; in nearly all there is a supply of drinking water; and in many there are conveniences for washing, brushing, etc.

If American trains only consumed their own smoke, they would be perfection ; as it is there is a very serious drawback to American railway traveling in hot weather. To reach your journey's end with collar, cuffs and shirt front, which had been clean a few hours earlier, reduced to smoke-stained, cinder-dust strewn clouts, is not a pleasant experience. The fault is one which might be easily corrected.

